# Data-driven identification of structural alerts for mitigating the risk of drug-induced human liver injuries

**DOI:** 10.1186/s13321-015-0053-y

**Published:** 2015-02-11

**Authors:** Ruifeng Liu, Xueping Yu, Anders Wallqvist

**Affiliations:** Department of Defense Biotechnology High Performance Computing Software Applications Institute, Telemedicine and Advanced Technology Research Center, U.S. Army Medical Research and Materiel Command, 2405 Whittier Drive, Frederick, MD 21702 USA

**Keywords:** Drug-induced liver injury, Hepatotoxicity, Structure alert, Bioactivation, Reactive metabolite

## Abstract

**Background:**

The use of structural alerts to de-prioritize compounds with undesirable features as drug candidates has been gaining in popularity. Hundreds of molecular structural moieties have been proposed as structural alerts. An emerging issue is that strict application of these alerts will result in a significant reduction of the chemistry space for new drug discovery, as more than half of the oral drugs on the market match at least one of the alerts. To mitigate this issue, we propose to apply a rigorous statistical analysis to derive/validate structural alerts before use.

**Method:**

To derive human liver toxicity structural alerts, we retrieved all small-molecule entries from LiverTox, a U.S. National Institutes of Health online resource for information on human liver injuries induced by prescription and over-the-counter drugs and dietary supplements. We classified the compounds into hepatotoxic, nonhepatotoxic, and possible hepatotoxic classes, and performed detailed statistical analyses to identify molecular structural fragments highly enriched in the hepatotoxic class beyond random distribution as structural alerts for human liver injuries.

**Results:**

We identified 12 molecular fragments present in multiple marketed drugs that one can consider as common “drug-like” fragments, yet they are strongly associated with drug-induced human liver injuries. Thus, these fragments may be considered as robust hepatotoxicity structural alerts suitable for use in drug discovery screening programs.

**Conclusions:**

The use of structural alerts has contributed to the identification of many compounds with potential toxicity issues in modern drug discovery. However, with a large number of structural alerts published to date without proper validation, application of these alerts may restrict the chemistry space and prevent discovery of valuable drugs. To mitigate this issue, we showed how to use statistical analyses to develop a small, robust, and broadly applicable set of structural alerts.

**Graphical abstract:**
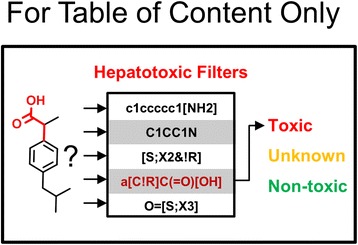
Hepatotoxic-specific filters for flagging high risk compounds.

**Electronic supplementary material:**

The online version of this article (doi:10.1186/s13321-015-0053-y) contains supplementary material, which is available to authorized users.

## Background

Despite significant progress in the field of chemical toxicology and drug safety assessment, accurate prediction of the occurrence of adverse drug reactions (ADRs) remains one of the major challenges in modern drug discovery [1]. The consequences cannot be overestimated, as surveys indicate that ADRs cost several billion dollars a year [2] and constitute one of the top 10 causes of death in the United States [3,4]. As the human liver metabolizes more than 90% of all prescription drugs [5] and is exposed to high concentrations of orally administered drugs and their metabolites [6], drug-induced liver injuries are the most frequently reported ADRs [7,8] and the most common reason for drug withdrawal [9]. To reduce the probability that drug candidates will have unwanted toxicities, many molecular structural moieties of high chemical reactivity, or those that can be transformed into moieties of high chemical reactivity by human enzymes (i.e., bioactivation), were proposed as structural alerts [10-12]. However, there was no publication specifically dedicated to the development of structural alerts for mitigating the risk of drug-induced human liver injuries until very recently [13]. On the other hand, over two thousand structural alerts for flagging various undesirable features of drug candidates have been assembled in the Online Chemical Database – a web-based resource at https://ochem.eu/. The assumption is that removing compounds with structural alerts from bioactivity screening libraries and short lists of drug candidates would reduce the risk of drug discovery and development failures.

However, there is a growing concern that some structural alerts might be too stringent and that strictly applying them would severely limit the chemical diversity needed to operate drug discovery programs. As pointed out by Stepan et al., nearly half of all new small-molecule drugs possess at least one structural alert, and some alerts are also present in the top-selling drugs [14]. Indeed, our profiling (using the web tool at https://ochem.eu/alerts/home.do) of 826 U.S. Food and Drug Administration (FDA)-approved oral drugs retrieved from DrugBank (http://www.drugbank.ca/) indicates that 514 (62.2%) of the drugs match reactive, unstable, or toxic structural alerts, and 414 (50.1%) of them match at least one of the idiosyncratic toxicity structural alerts of Kalgutkar et al. [11] If these alerts were strictly enforced, we would not have half of the oral drugs currently on the U.S. market! To prevent this, it is to crucial to develop structural alerts that are strongly associated with increased occurrences of chemical-induced toxicity in the therapeutic dose range, not merely those that may participate in a relevant bioactivation pathway but without clinical evidence of resulting human injuries, nor those that are only known to cause injuries in an animal model. The latter consideration stems from toxic endpoints being dose-dependent [14,15], and animal models tend to use doses higher than the equivalent human doses.

To demonstrate a strong association between a structural alert and a chemical-induced toxicity, the structural alert should occur significantly more in compounds positive for the toxicity than in compounds negative for the toxicity. Unfortunately, this is not always the case. For instance, in a recent paper by Hewitt et al., 16 structural moieties were flagged as structural alerts for human hepatotoxicity [13]. However, one of the alerts (alert 5 in the paper) is present in eight nonhepatotoxic and three hepatotoxic drugs. In addition, other alerts (alerts 1, 4, and 13) occur almost equally in the hepatotoxic and nonhepatotoxic drugs [13]. In our opinion, a strong association between a structural alert and a chemical-induced toxicity should be established by statistical analyses in order to provide a robust indication, as opposed to a casual association.

There are several hurdles in deriving meaningful human hepatotoxicity structural alerts. Perhaps the largest is the lack of a large and carefully curated human hepatotoxicity dataset. Information about idiosyncratic human liver ADRs is chiefly accumulated via reports from prescribing physicians after drugs received FDA approval. These ADRs typically occur in a small subset of the patient population and are not observed in relatively small, short-term clinical trials. Data from such sources are noisy because of reporting bias. For example, ADRs may be over-reported for a new or “untrusted” drug and under-reported for a “trusted” drug. In addition, many patients take multiple drugs for treating different and usually unrelated conditions. A liver adverse event could be induced by one of the drugs or by multiple drugs via synergistic drug-drug interactions [16]. Thus, establishing a causative relationship between a liver adverse event and a specific drug molecule is not trivial. Further complicating the matter is the lack of an established threshold on the severity of a liver adverse event for defining when a drug should be classified as hepatotoxic. For example, an adverse drug event may be under-reported by young and relatively healthy patients who perceive it as minor, whereas the same event might appear life-threatening to an older patient suffering from multiple health issues. To mitigate these challenges, some studies consider drugs that induce elevation of human liver enzymes as hepatotoxic. Other studies consider compounds that induce liver injuries in lab animals as hepatotoxic for humans. However, many safe and efficacious drugs induce transient elevations of human liver enzymes. The elevated levels may return to normal with continued therapy or shortly after completion of therapy, without apparent liver injury. Although animal models are commonly used in pre-clinical research, there are many examples of compounds that are safe in animal models or efficacious in an animal disease model, but toxic to a human or ineffective for treating a human disease.

Recently, the National Library of Medicine of the U.S. National Institutes of Health launched LiverTox, a database of ~700 medications associated with human liver injuries [17]. It provides evidence-based information related to liver injuries associated with prescription and over-the-counter drugs, herbal remedies, and dietary supplements. Carefully curated and reviewed by experts in multiple disciplines, the database constitutes a valuable resource for developing and validating structural alerts for drug-induced human liver injuries. Toward this goal, we retrieved from LiverTox all small-molecule entries with molecular structures. We augmented the dataset with drugs withdrawn from market and drugs with black-box warning labels due to acute human liver injuries. We used the expanded dataset to identify structural alerts that are present mostly in liver-toxic drugs, and their presence is unlikely due to random distribution.

## Results and discussion

**Figure 1:**
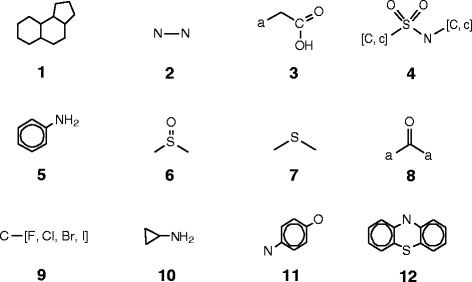
**Molecular structural moieties defined by the SMARTS in Table** 1 **.** Lowercase element symbols represent aromatic atoms of the element; the letter “a” matches any aromatic atom. Elements in square brackets match any of the elements in a molecule.

Table 1 shows the hepatotoxicity structural alerts derived in this study presented in the form of Smiles ARbitrary Target Specification (SMARTS) notations [18]. Also shown in Table 1 are the number of compounds that matched a structural alert in the three hepatotoxicity classes and the p-values calculated by the method described in the Methods. Figure 1 presents the structural moieties represented by the SMARTS notations, and Table 2 gives names of the drugs matched to these structural moieties. While most drugs in Table 2 match one of the structural alerts, a very small number of drugs match 2 or 3 different alerts. Details on these drugs and the structural alerts they match can be found in the Additional file 1 that is freely downloadable from the journal web site.

**Table 1:** **Structural alerts for human liver toxicity and their frequency of occurrence in each of the drug classes**

**Alert**	**SMARTS** ^**a**^	**Hepatotoxic** ^**b**^ **(178)**	**Possible hepatotoxic** ^**c**^ **(243)**	**Nonhepatotoxic** ^**d**^ **(186)**	**p-value** ^**e**^
1	C12CCCCC1C3C(CCC3)CC2	19	2	3	<0.0001
2	NN	14	13	0	<0.0001
3	a[C!R]C(=O)[OH]	11	7	0	0.0011
4	[#6]S(=O)(=O)N[#6]	18	16	3	0.0058
5	c1ccccc1[NH2]	7	4	0	0.013
6	O = [S;X3]	5	0	1	0.014
7	[S;X2&!R]	8	14	1	0.016
8	a[C!R](=O)a	10	4	1	0.029
9	C[F,Cl,Br,I]	21	23	7	0.039
10	C1CC1N	4	4	0	0.11
11	[O]c1ccc([N])cc1	5	4	1	0.25
12	N1c2ccccc2Sc2ccccc12	5	2	1	0.25

^a^SMiles ARbitrary Target Specification (SMARTS), a language for describing molecular patterns from Daylight Information Systems, Inc. (ref. [18]). ^b^Drugs known to cause clinically apparent acute human liver injuries; the total number is given in parentheses. ^c^Drugs that may have been linked to some reports of human liver injuries, but have not been convincingly established as causing these injuries in their therapeutic doses, or have not been widely used for an extended period of time and, therefore, lack sufficient clinical data for a reliable classification. The total number of these drugs is given in parentheses. ^d^Drugs that have been on the market for an extended period of time and are in widespread use, but have not been convincingly associated with clinically apparent acute human liver injuries. The total number of these drugs is given in parentheses. ^e^Probability for a structural alert to have a specific occurrence pattern across the three drug classes by chance.

**Table 2:** **Names and hepatotoxicity classes** ^**a**^ **of the drugs that matched the structural alerts defined in Table** 1

**Drug name**	**Class**	**Drug name**	**Class**	**Drug name**	**Class**	**Drug name**	**Class**
***Alert 1***		Ketoprofen	0	***Alert 7***		Efavirenz	1
Triamcinolone	1	Naproxen	0	Ebrotidine	1	Nilutamide	0
Prednisone	1	Tolmetin	0	Niperotidine	1	Fluoxetine	0
Prednisolone	1	***Alert 4***		Penicillamine	1	Fluvoxamine	0
Methylprednisolone	1	Zafirlukast	1	Nelfinavir	1	Sitagliptin	0
Hydrocortisone	1	Piroxicam	1	Imipenem	1	Flucytosine	0
Dexamethasone	1	Tipranavir	1	Meropenem	1	Fluorouracil	0
Cortisone	1	Delavirdine	1	Disulfiram	1	Nilotinib	0
Betamethasone	1	Glyburide	1	Azathioprine	1	Fluphenazine	0
Testosterone	1	Glipizide	1	Spironolactone	0	Celecoxib	0
Stanozolol	1	Glimepiride	1	Clindamycin	0	Clindamycin	0
Oxymetholone	1	Gliclazide	1	Ceftriaxone	0	Riluzole	0
Oxandrolone	1	Fosamprenavir	1	Ertapenem	0	Maraviroc	0
Norethandrolone	1	Amprenavir	1	Ranitidine	0	Chlorambucil	0
Nandrolone	1	Tolbutamide	1	Nizatidine	0	Cyclophosphamide	0
Methyltestosterone	1	Tolazamide	1	Famotidine	0	Lomustine	0
Methenolone	1	Chlorpropamide	1	Cimetidine	0	Melphalan	0
Methandienone	1	Acetohexamide	1	Thioridazine	0	Bendroflumethiazide	0
Fluoxymesterone	1	Ebrotidine	1	Pergolide	0	Methyclothiazide	0
Danazol	1	Sudoxicam	1	Montelukast	0	Polythiazide	0
Exemestane	0	Sulfasalazine	1	Polythiazide	0	Silodosin	0
Spironolactone	0	Sulfadiazine	1	Albendazole	0	Desflurane	0
Dutasteride	−1	Sulfamethoxazole	0	Captopril	0	Sevoflurane	0
Finasteride	−1	Meloxicam	0	Cefazolin	−1	Bicalutamide	0
Eplerenone	−1	Sildenafil	0	***Alert 8***		Capecitabine	−1
***Alert 2***		Darunavir	0	Bromfenac	1	Emtricitabine	−1
Diclofenac	1	Sulfadoxine	0	Tienilic acid	1	Mechlorethamine	−1
Pirprofen	1	Rosuvastatin	0	Zomepirac	1	Dutasteride	−1
Lumiracoxib	1	Sotalol	0	Clometacin	1	Colestipol	−1
Fenclozic acid	1	Chlorothiazide	0	Benziodarone	1	Quazepam	−1
Fenclofenac	1	Polythiazide	0	Amiodarone	1	Chloral hydrate	−1
Clometacin	1	Methyclothiazide	0	Benzbromarone	1	***Alert 10***	
Zomepirac	1	Hydrochlorothiazide	0	Tolcapone	1	Nevirapine	1
Ibufenac	1	Bendroflumethiazide	0	Fenofibrate	1	Trovafloxacin	1
Bromfenac	1	Sumatriptan	0	Mebendazole	0	Abacavir sulfate	1
Benoxaprofen	1	Naratriptan	0	Indomethacin	0	Ciprofloxacin	1
Alclofenac	1	Almotriptan	0	Ketoprofen	0	Saxagliptin	0
Fexofenadine	0	Bosentan	0	Tolmetin	0	Tranylcypromine	0
Ticarcillin	0	Probenecid	−1	Ketorolac	−1	Gemifloxacin	0
Ibuprofen	0	Torsemide	−1	***Alert 9***		Moxifloxacin	0
Indomethacin	0	Vardenafil	−1	Leflunomide	1	***Alert 11***	
Ketoprofen	0	***Alert 5***		Isoflurane	1	Amodiaquine	1
Naproxen	0	Sulfadiazine	1	Enflurane	1	Ketoconazole	1
Tolmetin	0	Bromfenac	1	Triamcinolone	1	Minocycline	1
***Alert 3***		Nomifensine	1	Dexamethasone	1	Sulfasalazine	1
Diclofenac	1	Amprenavir	1	Betamethasone	1	Posaconazole	1
Pirprofen	1	Fosamprenavir	1	Tipranavir	1	Acetaminophen	0
Lumiracoxib	1	Procainamide	1	Pantoprazole	1	Itraconazole	0
Fenclozic acid	1	Lenalidomide	1	Lansoprazole	1	Acebutolol	0
Fenclofenac	1	Sulfadoxine	0	Trifluoperazine	1	Lapatinib	0
Clometacin	1	Darunavir	0	Gemcitabine	1	Tigecycline	−1
Zomepirac	1	Dapsone	0	Floxuridine	1	***Alert 12***	
Ibufenac	1	Sulfamethoxazole	0	Mefloquine	1	Chlorpromazine	1
Bromfenac	1	***Alert 6***		Flecainide	1	Pipamazine	1
Benoxaprofen	1	Sulindac	1	Flutamide	1	Perphenazine	1
Alclofenac	1	Lansoprazole	1	Halothane	1	Prochlorperazine	1
Fexofenadine	0	Omeprazole	1	Fluoxymesterone	1	Trifluoperazine	1
Ticarcillin	0	Pantoprazole	1	Ifosfamide	1	Fluphenazine	0
Ibuprofen	0	Rabeprazole	1	Carmustine	1	Thioridazine	0
Indomethacin	0	Modafinil	−1	Tolrestat	1	Promethazine	−1

^a^Expanded LiverTox dataset compound classes: 1, hepatotoxic; −1, nonhepatotoxic ; 0, possible hepatotoxic.

Alert 1 is a fused tricyclic saturated hydrocarbon moiety that is shared by a class of steroids known to cause acute human liver injuries with prolonged use or overdose. In the expanded LiverTox dataset, 19 drugs with this moiety were found in the hepatotoxic class, 3 in the nonhepatotoxic class, and 2 in the possible hepatotoxic class. A recent study on the development of human liver toxicity structural alerts defined three individual and larger structural moieties as alerts for estrogen steroids, anabolic steroids, and glucocorticoid steroids [13]. Alert 1 is the maximum common substructure of the three alerts proposed in reference 13.

Alert 2 matches hydrazines. Fourteen drugs with this structural moiety were found in the hepatotoxic class, 13 in the possible hepatotoxic class, but zero in the nonhepatotoxic class. It has a p-value of less than 10^−4^, which signifies a relatively strong association between liver-related adverse events and this structural feature.

Alert 3, an arylacetic acid, is a high-profile hepatotoxic structural alert, as there were 11 compounds with this structural moiety in the hepatotoxic class, zero in the nonhepatotoxic class, and 7 in the possible hepatotoxic class. It should be noted that 10 of the 11 hepatotoxic drugs having this structural moiety had been withdrawn from the market due to ADRs, including severe and fatal drug-induced acute human liver injuries. As we mention in the Methods, most compounds in the possible hepatotoxic class were associated with a low number of liver ADR reports, but the causative relationship between the drugs and the reported liver ADRs have not been well established. Considering that 10 out of the 11 drugs having this structural moiety in the hepatotoxic class had been withdrawn from market, the observed human liver injuries associated with the 7 drugs in the possible hepatotoxic class have a high likelihood of being caused by the drugs. It may be just a matter of time for sufficient liver-injury reports to surface and for re-classification of the drugs into the hepatotoxic class.

Alert 4 is a sulfonamide moiety known to be associated with drugs that may cause human liver injuries [19]. This was corroborated by the expanded LiverTox dataset, as there were 18 drugs with this moiety in the hepatotoxic class, 15 in the possible hepatotoxic class, and only 3 in the nonhepatotoxic class. The p-value for the distribution of the compounds in the three hepatotoxicity classes is only 8.3 × 10^− 3^, indicating that this pattern is highly unlikely to occur by chance.

Many drugs with the sulfonamide group are safe and efficacious when administered at a relatively low dose and for a short duration. However, sulfonamides are linked to cases of acute liver failure and ranked in the top 10 causes of drug-induced, idiosyncratic fulminant hepatic failure [13]. Thus, to reduce the risk of drug-induced liver injuries, one should be aware of the hepatotoxicity liability associated with the sulfonamide group and consider replacing the structural moiety when feasible.

Alert 5 is the aniline moiety. Many compounds with this structural moiety are known to be mutagenic [20]. In the expanded LiverTox dataset, 7 compounds with this structural feature were found in the hepatotoxic class, 4 in the possible hepatotoxic class, and zero in the nonhepatotoxic class.

Alert 6 mainly occurs in a class of proton pump inhibitor drugs. Five of these drugs were found in the hepatotoxic class, and one in the nonhepatotoxic class.

Alert 7 is an acyclic bivalent sulfur moiety, a chemical group known to have a relatively high reactivity. Eight compounds with this structural moiety were found in the hepatotoxic class, 14 in the possible hepatotoxic class, and only 1 in the nonhepatotoxic class.

Alert 8 is an acyclic di-aryl ketone moiety. Ten compounds with this structural moiety were found in the hepatotoxic class, 1 in the nonhepatotoxic class, and 4 in the possible hepatotoxic class. Among the 10 drugs in the hepatotoxic class with this structural moiety, 5 were withdrawn from market due to severe and even fatal human liver injuries. Thus, Alert 8 is another structural moiety associated with an elevated liability of severe acute human liver injuries.

Alert 9 is a halogen atom bonded to a sp^3^ carbon. In this structural moiety, the halogen atoms are facile leaving groups in SN2 reactions and, therefore, this alert signifies a relatively high chemical reactivity. Twenty-one compounds matching this structural alert were found in the hepatotoxic class, 7 in the nonhepatotoxic class, and 23 in the possible hepatotoxic class, giving rise to a p-value of 3.9 × 10^− 2^.

Alert 10 matches a relatively small number of compounds in the expanded LiverTox dataset: 4 in the hepatotoxic class, 4 in the possible hepatotoxic class, and zero in the nonhepatotoxic class. The alert has a relatively high p-value of 0.11, partly as a result of a relatively small number of drugs (8) having this structural moiety.

Alert 11 is a para oxygen and nitrogen di-substituted benzene ring. It is known to form a quinoid structure upon bioactivation by liver enzymes, which may contribute to the potential hepatotoxic liability associated with the structural moiety. In the expanded LiverTox dataset, 5 drugs with this structural moiety were found in the hepatotoxic class, 4 in the possible hepatotoxic class, and only 1 was found in the nonhepatotoxic class.

Alert 12 is a fused tricyclic structural moiety found in some central nervous system drugs. Five drugs with this alert were in the hepatotoxic class, 2 in the possible hepatotoxic class, and 1 in the nonhepatotoxic class. Although the number of drugs with this structural moiety is relatively low, and the drugs distribute across all three classes, it is known that these drugs can induce acute intrahepatic cholestasis, steatosis, or hepatitis [21]. Proposed mechanisms of liver toxicity induced by these drugs include dissipation of the mitochondrial transmembrane potential and the inhibition of the electron transport chain [22,23].

In addition to the structural alerts described above, we also performed substructure searches using other structural alerts published in the literature [11,13]. However, some of the structural alerts were found in very few drugs in the expanded LiverTox dataset or were not present at all. They may be structural moieties associated with very high levels of toxicity, so that most compounds with these alerts failed to reach the market; or the moieties are not drug-like enough and therefore have a lower chance to become part of a drug. In either case, there were insufficient data in the expanded LiverTox dataset to evaluate these alerts.

## Conclusions

In summary, widespread use of structural alerts in drug discovery programs has inspired publication of thousands of structural alerts. Many of them have not been thoroughly validated with relevant data. Strict application of these alerts to remove compounds from bioactivity screening libraries and lists of drug development candidates may significantly lower the productivity of new drug discovery. To prevent this from happening, we propose to develop/validate structural alerts from relevant data with vigorous statistical analysis. As an example, we retrieved drug-induced human liver injury data from the recently launched LiverTox database and performed statistical analyses to identify structural moieties strongly associated with human liver injuries. A total of 12 such structural moieties were identified, and they can be used as human hepatotoxicity structural alerts to filter compound libraries and prioritize/profile drug candidates.

## Methods

We retrieved human liver ADR information for all entries in LiverTox in March 2014 via web access at http://livertox.nih.gov/. We then removed entries without chemical structures, such as some herbal extracts and vaccines. This gave us a list of 577 compounds. Each compound was annotated with a summary statement of reported human liver injuries, severity of the injuries, and a qualitative description of reporting frequencies. However, LiverTox does not include a categorical statement on whether a compound is hepatotoxic.

To classify the compounds into hepatotoxic and nonhepatotoxic groups, we initially followed the Drug-Induced Liver Injury Network’s five-point categorization of the likelihood that a medication is associated with drug-induced liver injuries [24]. The categories are described below.

Category A: The drug is well-known, well described, and frequently reported to cause either direct or idiosyncratic liver injury, and it has a characteristic signature; more than 50 cases, including case series, have been described.

Category B: The drug is reported and known to cause idiosyncratic liver injury and has a characteristic signature; between 12 and 50 cases, including small case series, have been described.

Category C: The drug is probably linked to idiosyncratic liver injury but has been reported infrequently, and no characteristic signature has been identified; the number of identified cases is less than 12, without a significant case series.

Category D: Single case reports have appeared implicating the drug, but fewer than three cases have been reported in the literature; no characteristic signature has been identified, and the case reports may not have been very convincing. Thus, these drugs can only be said to be possible hepatotoxins.

Category E: Despite extensive use, there is no evidence that the drug has caused liver injury. Single case reports may have been published, but they were largely unconvincing. These drugs are not believed to cause liver injury.

Category X: For drugs recently introduced or rarely used in clinical medicine, there may be inadequate information to place it in any of the five categories. Thus, this category is characterized as “unknown”.

Because counts of drug-induced liver injury reports were unavailable in LiverTox, we then implemented a slightly modified categorization scheme that does not rely on counting reports. We combined categories A and B into a hepatotoxic class; categories C, D, and X into a possible hepatotoxic class; and we left category E as a nonhepatotoxic class. That is, any compound described as a well-known cause, a cause, or a rare cause of clinically apparent acute human liver injuries was classified as hepatotoxic. We also classified as hepatotoxic some compounds that might have a lower count of liver injury reports but were associated with very severe and fatal liver injuries, because even a very small number of drug-induced liver injury cases that result in fatalities may trigger a mandatory withdrawal or a black-box warning label. In the end, we classified 150 compounds as hepatotoxic, 185 as nonhepatotoxic, and 242 as possible hepatotoxic.

As a valuable information resource for liver ADRs of current prescription and over-the-counter drugs, LiverTox does not contain information about some drugs that were withdrawn from the market due to drug-induced liver toxicity. Twenty-five such drugs were cited by Kalgutkar [4] and Stepan et al. [14]. We included them in the hepatotoxic class. In addition, Stepan et al. cited some drugs with black-box warning labels for their hepatotoxicity liability, and three of them were not in LiverTox. We also included the three drugs in the hepatotoxic class. Our final dataset has 178 hepatotoxic compounds, 185 nonhepatotoxic compounds, and 242 compounds with possible hepatotoxic. We call this dataset the expanded LiverTox dataset, and it is provided as supporting information.

We next performed substructure searches using previously proposed structural alerts for reactive, unstable, and toxic compounds [11-13] as queries. The substructure searches identified occurrence of these alerts in the compounds of the three hepatotoxicity classes. We then performed statistical analyses to evaluate the probability of the occurrence patterns of the alerts in the three hepatotoxicity classes by chance. Only patterns with a very low probability of chance occurrence (i.e., a low p-value) were considered evidence for valid hepatotoxicity structural alerts.

We calculated the p-values for the mutual information (MI) [25] between two classifications for each structural alert. The first classification divided the drugs into three groups: hepatotoxic, nonhepatotoxic, and possible hepatotoxic. The second classification divided the drugs into two groups: those that contained the structural alert and those that did not. Thus, we constructed a 2 × 3 matrix for each structural alert and computed the MI of this matrix. We performed 10,000 simulations by randomly distributing the drugs in the 2 × 3 matrix without changing the sums of each row or column and counted the fraction of generated matrices having MI values larger than or equal to those observed in the expanded LiverTox dataset. We used this fraction as the p-value for observing structural alerts of our three classification categories compared with random observations. We chose to run 10,000 simulations to ensure sufficient statistical reliability of the calculated p-values, as this choice allowed us to calculate p-values down to a lower limit of 0.0001 (1/10,000).

## Additional file

Additional file 1:
**Expanded LiverTox dataset comprising 178 hepatotoxic, 185 nonhepatotoxic, and 242 possible hepatotoxic compounds.**
